# Coping with Public and Private Face-to-Face and Cyber Victimization among Adolescents in Six Countries: Roles of Severity and Country

**DOI:** 10.3390/ijerph192114405

**Published:** 2022-11-03

**Authors:** Michelle F. Wright, Sebastian Wachs, Takuya Yanagida, Anna Ševčíková, Lenka Dědková, Fatih Bayraktar, Ikuko Aoyama, Shanmukh V. Kamble, Hana Macháčková, Zheng Li, Shruti Soudi, Li Lei, Chang Shu

**Affiliations:** 1 DePaul University, Chicago, IL 60614, USA; 2 Dublin City University, D09 Dublin, Ireland; 3 University of Potsdam, 14469 Potsdam, Germany; 4 University of Vienna, 1010 Wien, Austria; 5 Masaryk University, 60177 Brno, Czech Republic; 6 Eastern Mediterranean University, Famagusta 99628, Turkey; 7 Tsuru University, Tsuru, Yamanashi 402-8555, Japan; 8 Karnatak University, Dharwad, Karnataka 580003, India; 9 Beijing University of Technology, Beijing 100021, China; 10 Christ University, Bangalore, Karnataka 560029, India; 11 Renmin University of China, Beijing 100872, China

**Keywords:** coping, country, culture, victimization, severity, cyberbullying, bullying

## Abstract

This study investigated the role of medium (face-to-face, cyber) and publicity (public, private) in adolescents’ perceptions of severity and coping strategies (i.e., avoidant, ignoring, helplessness, social support seeking, retaliation) for victimization, while accounting for gender and cultural values. There were 3432 adolescents (ages 11–15, 49% girls) in this study; they were from China, Cyprus, the Czech Republic, India, Japan, and the United States. Adolescents completed questionnaires on individualism and collectivism, and ratings of coping strategies and severity for public face-to-face victimization, private face-to-face victimization, public cyber victimization, and private cyber victimization. Findings revealed similarities in adolescents’ coping strategies based on perceptions of severity, publicity, and medium for some coping strategies (i.e., social support seeking, retaliation) but differential associations for other coping strategies (i.e., avoidance, helplessness, ignoring). The results of this study are important for prevention and intervention efforts because they underscore the importance of teaching effective coping strategies to adolescents, and to consider how perceptions of severity, publicity, and medium might influence the implementation of these coping strategies.

## 1. Introduction

Adolescents’ experience of bullying is a global public health concern. Bullying is found in various cultures and it negatively impacts adolescents’ development [1,2,3,4]. Adolescents’ coping strategies fluctuate based on the publicity (whether public or private) and the specific type of bullying victimization, specifically face-to-face vs. cyber [5]. Public forms of bullying occur with others present, while private forms of bullying occur between the perpetrator and victim. Defined as being targeted with harassment, face-to-face bullying involves humiliation, embarrassment, intimidation, and/or threats, and cyberbullying involves these same behaviors, albeit carried out via information and communication technologies. Perceptions of severity influence on adolescents’ coping strategies is unknown. Coping strategies have significant impacts on adolescents’ psychological health, and it might be likely that medium and publicity could have a role in coping decisions. The objective of the present study was to examine the role of medium (face-to-face vs. cyber) and publicity (public vs. private) in perceptions of severity and coping strategies for peer victimization, while controlling for gender and cultural values, among adolescents in China, Cyprus, the Czech Republic, India, Japan, and the United States (U.S.).

### 1.1. Perceptions of Severity

Perceptions of severity might also be important for understanding more about adolescents’ coping strategies for peer victimization. In addition, perceptions of severity influence the likelihood of seeking help from others and when considering whether bullying is serious [6]. Some research has focused on adolescents’ perceptions of severity for different types of bullying situations. Smith and colleagues [4] found that picture and video clip bullying were perceived as more severe than traditional face-to-face bullying among adolescents (11–14 years old). In addition, face-to-face bullying was perceived to be more severe than phone call and text message bullying. Picture and video clip bullying are types of bullying that involve a greater audience, potentially increasing the publicity of these types of bullying, which might have made such bullying more salient and considered the worst type of bullying among adolescents.

Few studies have focused on publicity and type of bullying specifically when considering perceptions of severity. Sticca and Perren [7] found that greater perceptions of severity were found for public bullying situations than private bullying situations. These findings were further supported by studies involving interviews and focus groups [8]. Ultimately, it might be concluded that publicity and type of bullying have a role in perceptions of severity for peer victimization. Antibullying intervention programs usually focus on increasing adolescents’ knowledge concerning the severity of bullying situations to increase help-seeking behavior, ability to understand the impact of bullying, to promote provictim attitudes, and to reduce reinforcing bullies’ behaviors [9].

Given that perceptions of severity influence the desire for victims to seek help, it might be likely that such perceptions influence other coping strategies. Because perceptions of severity increase negative emotions related to bullying, adolescents might have difficulty employing adaptive coping strategies when they experience high negative emotions [10]. Focusing on the impact of perceptions of severity and coping strategies is important because coping strategies influence adolescents’ behavioral and psychological adjustment [11,12,13].

### 1.2. The Role of Culture and Gender in Coping Strategies for Peer Victimization

Reducing or removing the negative effects of stressful situations involves primary appraisal and secondary appraisal [14]. Primary appraisal is defined as ascribing meaning to a situation and is influenced by people’s values, commitments, and goals [15]. Secondary appraisal involves evaluating various coping strategies and making decisions about the outcomes associated with each strategy [14]. After possible outcomes are evaluated, a decision is made about the coping strategy or strategies enacted. In the literature, researchers usually focus on four main coping strategies, including retaliation (i.e., reactive response to the bully, seeking/getting revenge against the bully), social support (i.e., asking for help from friends, family, etc.), helplessness (i.e., not taking control over the situation), and ignoring [2].

Coping strategies might differ due to the publicity (public vs. private victimization) and type of peer victimization (face-to-face vs. cyber victimization). One study on this topic revealed that adolescents used more avoidant, social support, retaliation, helplessness, and ignoring coping strategies for public and face-to-face forms of victimization when compared to private and cyber forms of victimization [5]. Some research has focused on how the emotional impact of stressful situations influence coping strategies, which might shed some light on factors that influence coping strategies. Perceiving greater emotional impact for stressful situations influences the implementation and type of coping strategies for those situations [10]. The differential impact of emotions on face-to-face victimization and cyber victimization are unclear. Typically, adolescents perceive greater emotional impact in face-to-face victimization scenarios while other research suggest greater emotional impact of cyber victimization scenarios [4,16]. Mixed findings are indicative of different definitions and methodologies used to access victimization, although such findings might also indicate there are some potential differences in how adolescents perceive and deal with different types of victimization. Although they did not focus on coping strategies, Sticca and Perren [7] examined adolescents’ perceptions of harm for different types of victimization, whether public vs. private or face-to-face vs. cyber varied based on severity of harm. Adolescents perceived greater harm in public victimization than private victimization and for cyber victimization than face-to-face victimization, though effect sizes were small. Thus, based on the literature, coping strategies might change dependent on the type of victimization and where it occurs.

Culture also influences the type of coping strategies used to deal with peer victimization. Coping strategies are often implemented that match the cultural context [17]. Coping strategies that impact society, the broader environment, and consider others are usually utilized by people from collectivistic cultures [18,19]. In addition, people who typically employ coping strategies that suit their needs and focus on the self are often from individualistic cultures. In this research, Lam and Zane [20] investigated differences in Asian and European Americans’ coping strategies for peer victimization. Findings revealed that more direct control coping strategies were used by European Americans, while Asian Americans utilized more indirect control coping strategies. In sum, culture should be examined when examining coping strategies for peer victimization. Thus far, no research has focused on this topic.

Gender adds an interesting context when examining adolescents’ coping strategies for peer victimization. However, few studies have focused on gender differences in adolescents’ coping strategies for public and private forms of cyber victimization. In the literature, girls are more likely to ask for teacher help when dealing with peer victimization whereas boys are more likely to fight back [21]. Other research has found that girls are also more likely to ask friends for help and ask the aggressor why more so than boys, while boys are less likely to ask for help when compared to girls [22]. It is unclear whether culture might further impact gender differences in coping strategies for different types of peer victimization, including those that occur privately or publicly and those that occur offline vs. online.

### 1.3. The Present Study

**Figure 1:**
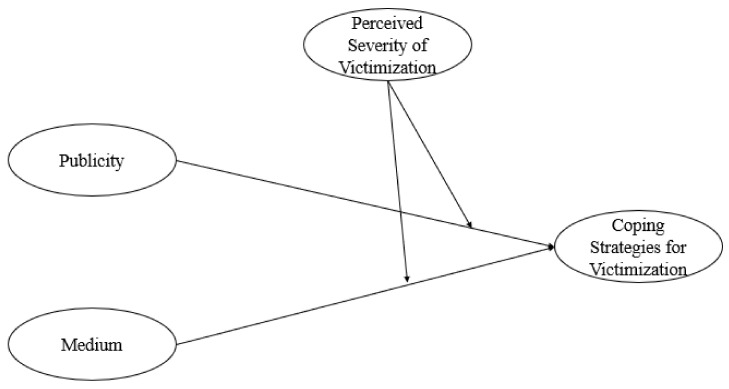
Model of the moderating effect of perceived severity of victimization in the relationships among publicity (public vs. private victimization), medium (face-to-face vs. cyber victimization), and coping strategies for victimization.

The major purpose of this study was to examine the role of publicity (public vs. private) and medium (face-to-face vs. cyber) in the associations between perceptions of severity and coping strategies among Chinese, Cypriot, Czech, Indian, Japanese, and U.S. adolescents. Gender and cultural values (i.e., individualism, collectivism) were controlled. China, Cypriot, India, and Japan were considered collectivistic; the Czech Republic and the U.S. were considered individualistic, with India also endorsing individualism too. Such research is important because the context of victimization can influence adolescents’ coping strategies and their subsequent adjustment [23]; however, contexts related to publicity and medium have not yet been investigated in the literature. Hypotheses were not created for this study because of the likelihood of interpreting multiple potential interactions (see Figure 1 for a model of the proposed relationships examined in this study). The following research questions were created for this study:

(1) What are the differences in adolescents’ severity and coping strategies between public and private face-to-face and cyber victimization?
(2) What are the interaction effects of country of origin, publicity, and medium on severity and attributions?

## 2. Methods

### 2.1. Participants

There were 3433 adolescents in 6th through 8th grades (age range 11–15 years old; 49.65% girls) included in this study. They were from six countries, including China (*n* = 674, 47.70% girls), Cyprus (*n* = 470; 49.80%), the Czech Republic (*n* = 537; 52.10% girls), India (*n* = 480; 46.50% girls), Japan (*n* = 460; 52.60%), and the U.S. (*n* = 812; 50.20% girls). No income or other demographic data were collected.

### 2.2. Procedures

Phone calls and emails were made or sent to school principals to introduce them to the study. Once school principals responded positively, announcements were then made to classrooms to describe the purpose of the study. Parental permission slips were also given to adolescents who then brought the slips home to their parents. Parent permission slips were returned to adolescents’ teachers. In Japan and the Czech Republic, there were modifications to the consent procedures such that school principals gave their consent for students to participate. Data were collected in school during the regular school day. Adolescents provided their assent to participate in the research prior to data collection. Only those adolescents who provided their assent (and had parent permission) participated in the study. All adolescents provided their assent. Questionnaires were randomized, completed via pen/pencil and paper, and administered in the following order: individualism and collectivism, and then the four peer victimization scenarios, with ratings of severity and coping strategies. Questionnaires were originally available in English, and later translated into the main language of the countries.

### 2.3. Measures

**Individualism and collectivism.** Cultural values were assessed using a 16-item questionnaire to assess adolescents’ individualism (eight items; e.g., My personal identity, independent of others, is very important to me) and collectivism (eight items; e.g., I feel good when I cooperate with others [24]). Adolescents rated the items on a scale of 1 (absolutely disagree) to 9 (absolutely agree). Items for individualism and collectivism were averaged separately to form separate scores for both variables, with Cronbach’s alphas ranging from 0.70 to 0.80 for individualism and 0.76 to 0.87 for collectivism.

**Severity and coping strategies for peer victimization.** Adolescents read four victimization scenarios, including (1) public face-to-face victimization, (2) public cyber victimization, (3) private face-to-face victimization, and (4) private cyber victimization. The following description was given for public face-to-face victimization: “A classmate says something really nasty and humiliating to you at school in front of everyone”. The statement of “at school in front of everyone” was underlined and bolded to make it more salient that this victimization occurred publicly and face-to-face. When a scenario was private, the “in front of everyone” was replaced with “but nobody is around to hear/see it”. When the medium of victimization varied, the “at school” was replaced with “online”. The designation of “online” indicated that cyber victimization was being referred to. After reading each scenario, adolescents answered a question about severity (i.e., How severe would you think this was [7]). Adolescents rated severity on a scale of 1 (not at all severe) to 4 (very severe).

After answering the question on severity, adolescents then rated ten coping strategy items [2]. Coping strategies included: social support (three items; e.g., I would talk to my friends about it), retaliation (one item; I would get back at him/her), avoidance (four items; e.g., I would forget about the situation), ignoring (one item; I would decide to ignore him/her), and helplessness (one item; I would do nothing because I do not want to make it worse). All items were rated on a scale of 1 (definitely WOULD not do) to 5 (definitely WOULD do). Cronbach’s alphas were 0.76 for social support and 0.71 for avoidance.

### 2.4. Analytic Plan and Missing Data

Using Mplus 7.4, multiple group multilevel models were performed to examine the study’s research questions. Level 1 included repeated measures which were nested within students at level 2 with the purpose of examining cross-country differences for coping strategies (i.e., social support, retaliation, avoidance, ignoring, helplessness) as the dependent variables [23,24]. Predictors were included at level 1, including publicity (0 = private, 1 = public), medium (0 = face-to-face, 1 = cyber), severity, two-way interactions (publicity and medium, publicity and severity, medium and severity), and a three-way interaction among publicity, medium, and severity. At level 2, gender, individualism, and collectivism were included. Centering was performed for severity, individualism, and collectivism using the grand mean for each country. The maximum likelihood estimators with robust errors using a sandwich estimator was used due to the non-independence of observations at the class level. Descriptive statistics for coping strategies were reported in a previous manuscript [25].

The missing at random assumption was taken into account by using the full information maximum likelihood estimate [26]. There were 270 incomplete records (0.9% of the data). Incomplete cases varied across the countries, with 47 from China, 109 from Cyprus, 87 from the Czech Republic, 5 from India, 21 from Japan, and 1 from the U.S.

## 3. Results

### 3.1. Control Variables: Gender, Individualism, and Collectivism

Chinese and Japanese boys were more likely to cope with victimization by using avoidance when compared to girls, while Czech girls were more likely to use avoidance coping in comparison to Czech boys (see Appendix A). Girls from China, India, and Japan were more likely to implement helplessness to cope with victimization, with opposite patterns for Cypriot boys and girls. Chinese girls were more likely to ignore victimization in comparison to boys. Chinese, Japanese, and U.S. boys were more likely to implement retaliation in response to victimization when compared to girls. The opposite pattern was found among Czech boys and girls. Girls were more likely to utilize social support seeking in China, India, Japan, and the U.S. when compared to boys. However, Cypriot and Czech boys were more likely to seek social support in response to victimization than girls.

Individualism was related positively to avoidance and ignoring among Japanese adolescents. Collectivism was associated negatively with avoidance among Chinese, Cypriot, and U.S. adolescents. Individualism was related positively to retaliation in all countries, whereas collectivism was associated negatively with retaliation among Chinese, Czech, Japanese, and U.S. adolescents. Among U.S. adolescents, individualism was negatively related to social support seeking. In addition, collectivism was related positively to social support seeking among Czech and Japanese adolescents. There were no relationships between helplessness or either cultural value.

### 3.2. Public vs. Private Victimization

Avoidance was used more for public victimization than private victimization among Chinese, Czech, Japanese, and U.S. adolescents. In addition, Japanese adolescents reported using helplessness more for public victimization in comparison to private victimization. Ignoring and social support seeking were implemented more by all adolescents, except Indian adolescents for ignoring, for public victimization when compared to private victimization. There were no differences in the use of retaliation for public vs. private forms of victimization.

### 3.3. Face-to-Face vs. Cyber Victimization

U.S. adolescents reported that they would use avoidance more for face-to-face victimization than cyber victimization. Chinese and Cypriot adolescents indicated that they would use ignoring more for cyber victimization than face-to-face victimization. Chinese, Czech, and U.S. adolescents utilized social support for face-to-face victimization more so than cyber victimization. There were no differences for helplessness and retaliation coping strategies.

### 3.4. Severity

Severity was negatively associated with avoidance for Chinese adolescents, although severity and avoidance were positively related for Cypriot, Czech, and U.S. adolescents. For Czech and U.S. adolescents, severity was associated positively with helplessness. In addition, severity was related negatively to ignoring for Cypriot adolescents, but positively for U.S. adolescents. For all adolescents, except Indian adolescents, severity was associated positively with social support seeking. Severity was unrelated to retaliation coping strategy.

### 3.5. Two-Way Interactions

Chinese adolescents used the avoidance coping strategy more for public face-to-face victimization than for private and cyber victimization. Similar patterns were found for Japanese adolescents and helplessness, as well as for ignoring among Indian and Japanese adolescents, retaliation among Czech and Japanese adolescents, retaliation among Czech and Japanese adolescents, and social support seeking among Chinese adolescents.

U.S. adolescents used more of the avoidance and helplessness coping strategies when they perceived more severity for face-to-face victimization than cyber victimization. Similar patterns were found for ignoring among Indian and Japanese adolescents as well as social support seeking and Japanese adolescents. Among Indian adolescents, they used more retaliation when they perceived more severity for cyber victimization when compared to face-to-face victimization.

Perception of severity was greater for private victimization than public victimization among Chinese adolescents when they used the avoidance coping strategy. Opposite findings were found for avoidance and retaliation among Czech adolescents, as well as social support seeking and Japanese adolescents. There was no interaction between medium and severity for the ignoring coping strategy.

### 3.6. Three-Way Interactions

There were no significant three-way interactions for avoidance, helplessness, and ignoring. Indian adolescents used the retaliation coping strategy more for private forms of cyber victimization at all levels of severity (low, average, and high), but not for public cyber victimization or face-to-face victimization. Japanese adolescents used social support seeking coping strategy more for public forms of face-to-face victimization at average and high levels of severity, but not for low levels, private face-to-face victimization, and cyber victimization.

## 4. Discussion

The purpose of the study was to investigate the roles of publicity (private, public) and medium (face-to-face, cyber) in the associations between perceptions of severity and coping strategies for victimization among adolescents from China, Cyprus, the Czech Republic, India, Japan, and the U.S, while controlling for gender, individualism, and collectivism. Findings revealed similarities in adolescents’ coping strategies based on perceptions of severity, publicity, and medium for some coping strategies (i.e., social support seeking, retaliation) but differential associations for others (i.e., avoidance, helplessness, ignoring). Such results highlight the importance of examining multiple contexts when assessing coping strategies. Follow-up research might aim to examine the relationships investigated in this study with longitudinal design, consider similar and differential impacts on future adjustment outcomes, and whether there are risk typologies among adolescents for future bullying and victimization. It is imperative that intervention and prevention efforts be developed with consideration to the role of medium, publicity, and severity in adolescents coping strategies, and for such programs to be culturally sensitive, given some of the divergent findings identified in this study. Focusing on helping adolescents identify and utilize effective coping strategies is important because such strategies have the potential to reduce risk and make them less vulnerable to repeated victimization and negative adjustment outcomes [12,27,28,29,30,31,32].

The most consistent finding from the present study was the social support seeking coping strategy. Adolescents from all countries relied more on this coping strategy for public victimization and they used greater levels of this coping strategy at higher perceptions of severity. Such a finding indicates the universality of social support seeking, especially when adolescents perceive victimization as severe. Available research has documented the use of social support in multiple countries and has consistently revealed the positive effects of utilizing this coping strategy on adolescents’ mental health [31,32,33,34]. Social support seeking is an effective coping strategy that mitigates negative adjustment outcomes and reduces vulnerability to and risk of engaging in victimization [35,36,37,38,39]. Our findings indicate the prominence of social support seeking in adolescents’ coping strategies repertoire and underscore the important function of this coping strategy for adolescents included in this study, regardless of their country of origin. A unique finding for the social support seeking coping strategy involves Japanese adolescents. They differentiated more so than other adolescents when choosing to use social support seeking in specific types of victimization situations. In particular, they were more likely to use this coping strategy for public face-to-face victimization than other types of victimization, especially when they perceived average to high levels of severity. Such a unique finding highlights further the importance of considering culturally sensitive intervention and prevention programs.

The next consistent finding was the retaliation coping strategy, with results revealing that adolescents, except for Indian adolescents, relied more on this coping strategy for public victimization than private victimization. Public forms of victimization might increase adolescents’ embarrassment, which could lead to seeking revenge against perpetrators. A further important consideration for future research, although not investigated in this study, might be to consider how adolescents choose to retaliate. For instance, adolescents might choose to retaliate more publicly in an effort to reduce negative emotions associated with public victimization [6,7,40]. Retaliating publicly might potentially help adolescents “save face” when they experience public victimization, as such victimization might increase negative emotions, especially embarrassment and anger [7,40]. Interestingly, severity did not predict the retaliation coping strategy. Such a finding is incredibly surprising as it might be expected that retaliation could be a coping strategy to use when adolescents perceive greater severity. We did not find support for this proposal, although we did find significant moderation effects for severity, publicity, and medium. A potential explanation might be that retaliation is less tied to adolescents’ perceptions of severity for victimization and more to the public nature of victimization and where it occurs (offline vs. online). Consistent with this premise is our finding that Czech adolescents utilized more retaliation when they perceived greater severity in public face-to-face victimization than private and cyber forms of victimization. For Indian adolescents, they were much more likely to respond with retaliation when their perceptions of severity were greater for private cyber victimization.

The results for avoidance, ignoring, and helplessness coping strategies were not as consistent across country of origin as the coping strategies of social support seeking and retaliation, although consistencies were generally found for publicity, medium, and severity. Avoidance coping was used more by Chinese adolescents for face-to-face victimization than other types of victimization, with similar patterns found for helplessness and ignoring among Japanese adolescents and ignoring for Indian adolescents. U.S. adolescents used avoidance and helplessness more in face-to-face victimization when they perceived greater severity. The coping strategy associations varied among adolescents from different countries but not the type of victimization, whether public or private and face-to-face or cyber. Such findings are consistent with our proposal that public face-to-face victimization might engender negative emotions more so than private forms, and such emotions might increase the use of coping strategies for public victimization [6,7,40]. Therefore, adolescents’ utilization of coping strategies for public victimization could help mitigate negative emotions, and negative emotions might increase negative adjustment outcomes. More research attention should be given to this proposal in future studies.

We cannot identify longitudinal associations among coping strategies and severity nor temporal ordering of these relationships, due to the cross-sectional nature of this study. Follow-up research should be conducted to explore potential changes over time in the role of publicity and medium in the longitudinal relationships among coping strategies and severity, and whether culture influences such relationships. Such research should also focus on *actual* coping strategies to better understand how context of victimization influences these coping strategies. It is also important for such research to investigate age differences in these associations. We focused exclusively on early adolescents and the findings of this present research might not apply to other populations. A focus of future research might be to explore whether perceptions of severity change as children begin to utilize information and communication technologies more autonomously and as peers become increasingly important. Such developmental changes might alter perceptions of public victimization when peers are more salient; children might be much more embarrassed when their peers are perceived as more important than when peers are perceived less important. Coping strategies are associated with future adjustment outcomes and behaviors [36,37,38,39,40]. The present research did not examine such outcomes and future research should address this aim. It might be likely that publicity, medium, and severity might alter adolescents’ adjustment outcomes, bullying, and victimization. We did not directly compare the countries in this study, given the exploratory nature of the study. The lack of literature made it difficult to craft specific hypotheses to guide the study. Follow-up research might consider direct comparisons among the countries that might be easier to hypothesize with our study’s findings.

## 5. Conclusions

It is important to understand the role of medium, publicity, and severity in adolescents’ coping strategies, and the need to also consider adolescents’ country of origin. Overall, the findings indicate consistencies for social support seeking and retaliation coping strategies among adolescents in China, Cyprus, the Czech Republic, India, Japan, and the U.S., with divergent findings for avoidance, ignoring, and helplessness.

Our findings indicate the importance of designing bullying prevention and intervention programs which are also mindful of the role of medium, publicity, and adolescents’ perceptions of severity in adolescents’ coping strategies. Some of these programs should implement curriculum sensitive to adolescents in some countries, as we found some differential associations for coping strategies.

## Data Availability

The data presented in this study are available on request from the corresponding author. The data are not publicly available due to privacy.

## Appendix A

**Table A1:** Victimization.

	China	Cyprus	Czech Republic	India	Japan	U.S.
	*β*	*β*	*β*	*β*	*β*	*β*
**Avoidance**						
Gender	***−0.10*** **	−0.06	***0.15*** ***	0.08	***−0.19*** ***	−0.05
Individualism	−0.01	0.04	−0.01	0.02	***0.08* * **	0.01
Collectivism	***−0.03*** *	***−0.07*** ***	0.03	−0.03	−0.04	***−0.02*** *
Pubic vs. Private	***0.16*** ***	0.06	***0.13*** ***	−0.09	***0.09*** ***	***0.10*** ***
F2F Vic vs. Cy Vic	−0.02	0.01	−0.04	0.03	−0.01	***−0.08*** *
Sev	***−0.09*** *	***0.10*** *	***0.16*** ***	0.06	0.07	***0.14*** ***
Med × Pub	***−0.09*** ***	−0.02	0.01	−0.01	−0.05	−0.01
Med × Sev	−0.04	−0.06	0.02	0.08	−0.01	***−0.07*** *
Pub × Sev	−0.01	−0.08	−0.02	0.02	−0.06	−0.05
Med × Pub × Sev	0.05	0.09	0.01	−0.06	0.05	0.07
**Helplessness**						
Gender	***−0.13*** **	***0.11*** *	0.05	***−0.11*** ***	***−0.27*** ***	−0.09
Individualism	0.01	−0.07	−0.01	−0.04	0.06	0.01
Collectivism	−0.02	0.03	−0.04	0.04	−0.04	−0.02
Pubic vs. Private	0.03	0.05	0.04	−0.02	***0.16 *** ***	−0.02
F2F Vic vs. Cy Vic	0.01	0.01	−0.02	0.01	0.01	−0.01
Sev	−0.05	−0.06	***0.14 *** ***	0.04	−0.01	***0.12*** **
Med × Pub	−0.01	−0.03	0.01	−0.02	***−0.12*** ***	−0.02
Med × Sev	0.03	0.08	−0.02	−0.02	−0.04	***−0.09*** *
Pub × Sev	***0.08*** *	0.05	***−0.09*** *	−0.06	−0.02	−0.09
Med × Pub × Sev	−0.06	−0.06	0.05	0.04	0.05	0.10
**Ignoring**						
Gender	***−0.11*** **	0.02	0.06	−0.02	0.09	−0.03
Individualism	***0.10*** **	0.05	0.06	0.06	***0.24*** ***	−0.06
Collectivism	0.14	0.06	0.04	0.13	−0.11	0.07
Pubic vs. Private	***0.10*** ***	***0.09*** ***	***0.12*** ***	0.04	***0.12*** ***	***0.17*** ***
F2F Vic vs. Cy Vic	***−0.06*** **	***−0.07*** ***	−0.01	−0.01	−0.01	0.01
Sev	0.09	***−0.09*** ***	−*0.03*	0.05	0.08	***0.10*** **
Med × Pub	−0.03	0.01	−*0.06*	***−0.09*** *	***−0.06*** *	−0.04
Med × Sev	−0.02	−0.01	−0.01	0.05	−0.02	0.02
Pub × Sev	−0.02	−0.03	−0.07	−0.01	−0.07	−0.09
Med × Pub × Sev	0.02	−0.01	0.04	−0.01	0.07	−0.01
**Retaliation**						
Gender	***0.10*** ***	−0.09	***−0.12*** *	0.10	***0.15*** **	***0.14 *** ***
Individualism	***0.17*** ***	***0.27*** ***	***0.18*** **	***0.23*** **	***0.32*** ***	***0.13*** ***
Collectivism	***−0.26*** ***	−0.05	***−0.12*** *	−0.10	***−0.23*** **	***−0.26*** ***
Pubic vs. Private	***0.04*** *	***0.09*** *	***0.06*** *	0.04	***0.08*** **	***0.12*** ***
F2F Vic vs. Cy Vic	0.01	−0.01	−0.01	0.04	0.03	−0.04
Sev	0.09	−0.03	−0.02	0.01	0.08	−0.04
Med × Pub	−0.04	−0.01	***−0.09*** *	−0.03	***−0.05*** *	−0.01
Med × Sev	−0.02	0.09	0.02	***0.15*** ***	0.01	0.02
Pub × Sev	0.02	−0.04	***−0.09*** *	0.05	−0.08	−0.03
Med × Pub × Sev	0.01	0.03	−0.02	***−0.20*** ***	0.04	0.02
**Social Support**						
Gender	***−0.09*** *	***0.16*** ***	***0.15*** **	***−0.18*** ***	***−0.12*** *	***−0.09*** *
Individualism	0.01	−0.02	−0.06	0.06	−0.09	***−0.08*** *
Collectivism	0.07	0.10	***0.13*** *	0.04	***0.37*** ***	0.09
Pubic vs. Private	***0.15*** ***	***0.12*** ***	***0.16*** **	***0.12*** ***	***0.06*** *	***0.15*** ***
F2F Vic vs. Cy Vic	***−0.06*** **	−0.02	***−0.09*** ***	−0.01	0.04	***−0.08*** *
Sev	***0.17*** ***	***0.15*** *	***0.25*** ***	−0.02	***0.38*** ***	***0.13*** *
Med × Pub	***−0.09*** ***	0.01	0.03	−0.08	−0.04	0.02
Med × Sev	0.01	−0.01	−0.01	0.09	***−0.06*** *	0.08
Pub × Sev	−0.03	−0.03	−0.08	0.06	***−0.13*** ***	−0.05
Med × Pub × Sev	−0.01	−0.02	0.06	−0.05	***0.09*** *	−0.08

Note. F2F = Face-to-face; Vic = Victimization; Cy = Cyber; Sev = Severity; Med = medium (face-to-face, cyber); Pub = publicity (public, private). Bolded and italicized numbers indicate statistically significant findings. * *p* < 0.05. ** *p* < 0.01. *** *p* < 0.001.
